# Two Pfam protein families characterized by a crystal structure of protein lpg2210 from *Legionella pneumophila*

**DOI:** 10.1186/1471-2105-14-265

**Published:** 2013-09-03

**Authors:** Penelope Coggill, Ruth Y Eberhardt, Robert D Finn, Yuanyuan Chang, Lukasz Jaroszewski, Adam Godzik, Debanu Das, Qingping Xu, Herbert L Axelrod, L Aravind, Alexey G Murzin, Alex Bateman

**Affiliations:** 1 Wellcome Trust Sanger Institute, Wellcome Trust Genome Campus, Hinxton, Cambridgeshire CB10 1SA, UK; 2 European Molecular Biology Laboratory, European Bioinformatics Institute, Wellcome Trust Genome Campus, Hinxton, Cambridgeshire CB10 1SD, UK; 3 Howard Hughes Medical Institute, Janelia Farm Research Campus, 19700 Helix Drive, Ashburn VA 20147, USA; 4 Program on Bioinformatics and Systems Biology, Sanford-Burnham Medical Research Institute, La Jolla, CA 92037, USA; 5 Joint Center for Structural Genomics, SLAC National Accelerator Laboratory, Menlo Park, CA 94025, USA; 6 Stanford Synchrotron Radiation Lightsource, SLAC National Accelerator Laboratory, Menlo Park, CA 94025, USA; 7 National Center for Biotechnology Information, National Library of Medicine, Building 38A, Bethesda, MD 20894, USA; 8 MRC Laboratory of Molecular Biology, Francis Crick Avenue, Cambridge Biomedical Campus, Cambridge CB2 0QH, UK

**Keywords:** Domain of unknown function, Protein family, Protein structure, DUF4424, YARHG domain, Sequence analysis

## Abstract

**Background:**

Every genome contains a large number of uncharacterized proteins that may encode entirely novel biological systems. Many of these uncharacterized proteins fall into related sequence families. By applying sequence and structural analysis we hope to provide insight into novel biology.

**Results:**

We analyze a previously uncharacterized Pfam protein family called DUF4424 [Pfam:PF14415]. The recently solved three-dimensional structure of the protein lpg2210 from *Legionella pneumophila* provides the first structural information pertaining to this family. This protein additionally includes the first representative structure of another Pfam family called the YARHG domain [Pfam:PF13308]. The Pfam family DUF4424 adopts a 19-stranded beta-sandwich fold that shows similarity to the N-terminal domain of leukotriene A-4 hydrolase. The YARHG domain forms an all-helical domain at the C-terminus. Structure analysis allows us to recognize distant similarities between the DUF4424 domain and individual domains of M1 aminopeptidases and tricorn proteases, which form massive proteasome-like capsids in both archaea and bacteria.

**Conclusions:**

Based on our analyses we hypothesize that the DUF4424 domain may have a role in forming large, multi-component enzyme complexes. We suggest that the YARGH domain may play a role in binding a moiety in proximity with peptidoglycan, such as a hydrophobic outer membrane lipid or lipopolysaccharide.

## Background

A significant percentage of proteins encoded by all known genomes consist of uncharacterized proteins that have never been studied experimentally and do not show significant sequence similarity to any known proteins. A frequent first step in the analysis of such proteins is their classification into protein families. Many research groups are focusing on the identification and definition of new protein families that are then deposited into protein family databases and used for annotation of proteins by resources such as UniProtKB. Some protein families consist entirely of uncharacterized proteins, and therefore are typically defined as domains of unknown function (DUF) or uncharacterized protein families (UPFs). The Pfam database now contains a large collection of these families [1]. In this work we have analyzed the DUF4424 family [Pfam:PF14415] and the YARHG domain [2] [Pfam:PF13308]. The YARHG domain is also experimentally uncharacterized, but it was named for its highly conserved characteristic motif found in many of the sequences.

In a synergistic effort, the NIH Protein Structure Initiative (PSI) centers are systematically targeting uncharacterized proteins with the goal of providing structural information for a significant portion of the protein universe, often using Pfam for guidance in target selection. In this instance, Joint Center for Structural Genomics (JCSG) has solved the crystal structure of a hypothetical protein (lpg2210) [UniProtKB:Q5ZTF2] encoded in the genome of *L. pneumophila* subsp. Pneumophila str. Philadelphia 1 as a representative of the DUF4424 family, and deposited the coordinates in the Protein Data Bank as [PDB:4g2a]. *L. pneumophila* invades and replicates within human monocytes and alveolar macrophages in humans, and also within Amoebae, and is the established causative agent of legionellosis or Legionnaires’ disease.

Little is known about the lpg2210 protein. In a gene expression study, *lpg2210* was found to be induced in the post-exponential growth phase, when *L. pneumophila* is known to express a variety of virulence factors *in vitro*[3]. Thus, it is plausible that lpg2210 and other members of this family, all of which have a signal sequence and are secreted proteins, may play a role in virulence.

## Results, Methods and Discussion

### Overall structure

**Figure 1:**
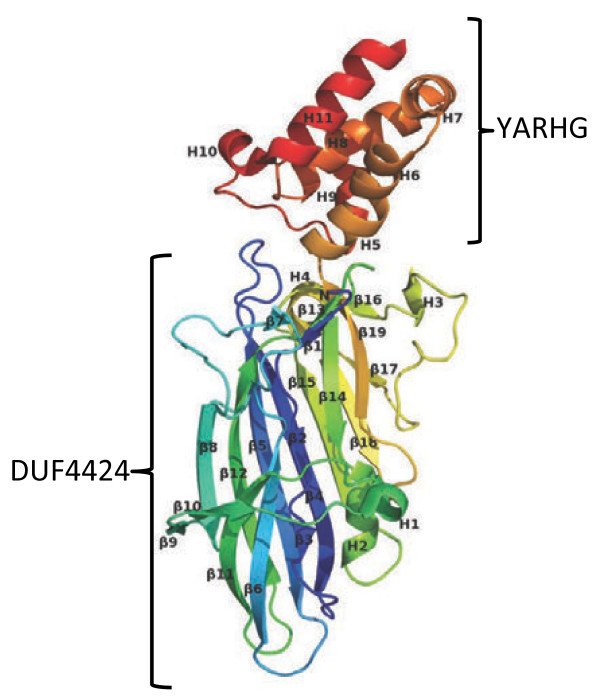
**A ribbon representation of the structure of the protein lpg2210 from*Legionella pneumophila.*** The structure of lpg2210 is colored in rainbow colors from the N-terminus in blue to the C-terminus in red. The N-terminus contains the DUF4424 domain; the C-terminus is the YARHG domain.

The crystal structure of lpg2210 from *L. pneumophila* subsp.pneumophila str philadelphia 1 was determined by three-wavelength MAD phasing at 2.33 Å resolution. Full details of data collection, model, and refinement statistics can be found in the Additional file 1. This protein contains two domains: an N-terminal DUF4424 domain that is mainly composed of a 19-stranded beta-sandwich fold and a YARHG domain that consists of a four-helical bundle in its C-terminal. The expressed protein contained a single N-terminal glycine (Gly 0) that remains after cleavage of the expression and purification tag, followed by residues 29–349 of the full-length protein. The asymmetric unit consists of one molecule of lpg2210. The final model includes residues Asn29 - Lys349, 14 sulphate molecules, and 160 water molecules. Electron density was disordered for Gly 0. The Matthews coefficient V_m_ is 2.23 Å^3^/Da and the estimated solvent content is 44.9%. The Ramachandran plot produced by Molprobity shows that 97.8% of the residues are in favored regions with no Ramachandran outliers. The protomer is composed of 19 β-strands, three β-sheets, six α-helices, five 3_10_-helices, 18 β-turns, two γ-turns, and one disulphide bond (Figure 1). PISA results suggest that the monomer may be the natural oligomerization state (http://www.ebi.ac.uk/msd-srv/prot_int/).

### Structure comparison

**Figure 2:**
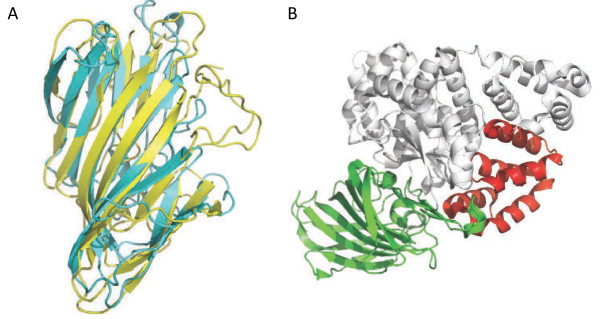
**Ribbon representation of structural alignment of the N-termini of human LTA4H and lpg2210. (A)** The N-terminal domains of human leukotriene A4-hydrolase [PDB:3fun] is shown in cyan and lpg2210 protein from *L. pneumophila* [PDB:4g2a] is shown in yellow. The alignment has 165 equivalent positions with an RMSD of 3.13 Å, and a P-value of 5.15e-04 determined using FATCAT [7] with a 5.2% sequence identity. **(B)** The arrangements of the N- and C-terminal domains in human leukotriene A4 hydrolase [PDB:3fun] is reminiscent of that seen in lpg2210. The central, catalytic domain in the human leukotriene A4 hydrolase (gray in the figure) is missing in lpg2210. The C-terminal, helical domain of LTA4H has a four helical bundle structure, with no statistically significant similarity to the lpg2210 YARHG domain.

To learn more about the potential function of the DUF4424 and YARHG domains we carried out structure comparisons of each domain against the PDB database using the DALI server [4]. The YARHG domain comparison yielded no significant similarities to any other structure. The DUF4424 domain search yielded the highest scoring alignments to the N-terminal region of leukotriene A-4 hydrolase (LTA4H) [PDB:3fun], with a significant Z score of 11.3 (Figure 2A), and to the N-terminus of a tricorn protease-interacting factor F3 [PDB:1z5h] with a Z score of 10.9. In both these cases this N-terminal domain is playing an auxiliary role in assisting the catalytic core, possibly by binding substrate [5]. SCOP [6] classifies the first subunit of [PDB:3fun] into an N-terminal region of two beta-sandwiches of similar topologies fused together into a single three beta-sheet domain, the second domain as a central catalytic region that is a catalytic metallopeptidase (“zincin”), and the third C-terminal helical region as part of the ARM superfamily. The organization of the first and third domains of LTA4H is reminiscent of the orientation of the two domains present in lpg2210, although only the first beta-sandwich domain is structurally similar. The YARHG domain is an all-helical domain that is structurally unlike the LTA4H C-terminal domain (Figure 2B). It is possible that the lpg2210 protein binds to another enzymatic domain that is analogous to the LTA4H metallopeptidase domain. According to the three-dimensional structure analysis of LTA4H, the N-terminal domain of this enzyme contains a large concave surface exposed to the solvent (Figure 2A) that could participate in the recognition of specific substrates [5]. It is possible that the equivalent surface on DUF4424 might also participate in substrate-recognition.

**Figure 3:**
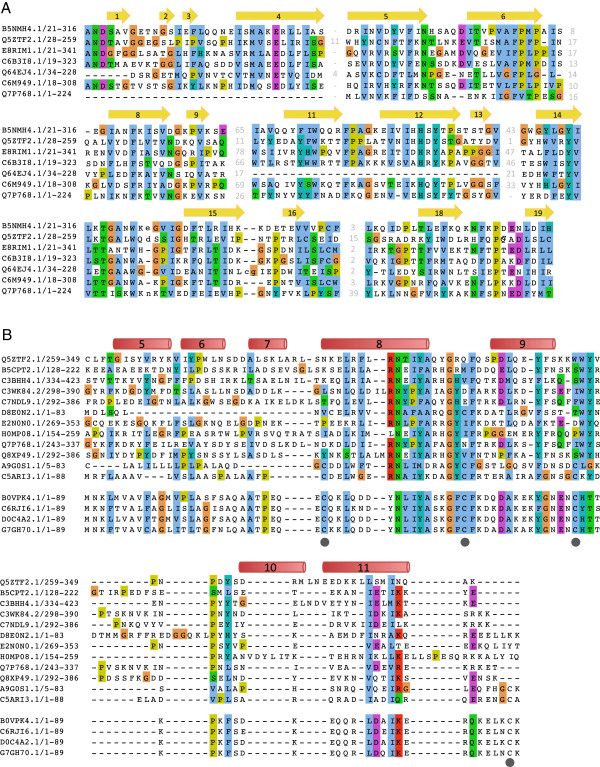
**Multiple sequence alignments of representative sequences in the DUF4424 and YARHG families.** The secondary structure of [PDB:4g2a], which corresponds to [UniProtKB:Q5ZTF2] is shown above the alignment, with alpha helices colored red and beta strands colored yellow. The secondary structure elements are numbered as in Figure 1. **(A)** Multiple sequence alignment of representative DUF4424 domain sequences. Sequences were chosen based on both domain architecture and taxonomic distribution. The following sequences contain a C-terminal YARHG domain: [UniProtKB:Q5ZTF2] (*Legionella pneumophila*), [UniProtKB:Q7P768] (*Fusobacterium nucleatum*). The following sequences all from different Proteobacterial classes contain only the DUF4424 domain: [UniProtKB:C6B3I8] (*Rhizobium leguminosarum* bv. trifolii), [UniProtKB:C6M949] (*Neisseria sicca*), [UniProtKB:E8RIM1] (*Desulfobulbus propionicus*), [UniProtKB:B5NMH4] (*Salmonella enterica*). [UniProtKB:Q64EJ4] contains a C-terminal CARDB domain (Archaea metagenomics). **(B)** Multiple sequence alignment of representative YARHG domains. The alignment has been split to show examples of the classical YARHG at the top and the YASKG subfamily at the bottom. The classical YARHG representatives are: [UniProtKB:B5CPT2] (*Ruminococcus lactaris ATCC 29176*), [UniProtKB:C3BHH4] (*Bacillus pseudomycoides*), [UniProtKB:C3WK84] (*Fusobacterium sp. 2_1_31*), [UniProtKB:C7NDL9] (*Leptotrichia buccalis*), [UniProtKB:D8E0N2] (*Prevotella bryantii*), [UniProtKB:E2N0N0] (*Capnocytophaga sputigena*), [UniProtKB:H0MP08] (*Salmonella enterica subsp. enterica serovar Montevideo str*), [UniProtKB:Q7P768] (*Fusobacterium nucleatum subsp. vincentii*), [UniProtKB:Q8XP49] (*Clostridium perfringens*), [UniProtKB:A9G0S1] (*Phaeobacter gallaeciensis*) The YASKG subfamily contains four conserved cysteine residues, that are marked by a gray circle below the column. The sequences shown for this subfamily are: [UniProtKB:B0VPK4] (*Acinetobacter baumannii*), [UniProtKB:C6RJI6] (*Acinetobacter radioresistens*), [UniProtKB:D0C4A2] (*Acinetobacter sp. RUH2624*), [UniProtKB:G7GH70] (*Acinetobacter sp. NBRC 100985*).

Although there were no structural similarities of the YARHG domain to any other structure, the structure is informative with respect to features of the domain itself. The YARHG domain family contains a subfamily, called YASKG that carries four conserved cysteine residues suggested to form two disulphide bridges (Figure 3B). The structure of the YARHG domain now allows us to evaluate this hypothesis and suggest a plausible bonding-pattern for the cysteines. The YASKG subfamily domains are relatively short, and carry only the YARHG domain with no other associated domains; these proteins are approximately 90 residues in length. The predicted positions of the cysteines on the [PDB:4g2a] version of YARHG indicate that only one pair share spatial proximity and could form a disulphide bridge. Cysteine 1 can potentially bond with cysteine 4. If cysteine 2 bonded with cysteine 3 it would induce significant structural rearrangements due to their distances from each other. The conformation of the shorter domains will be partly stabilized by the disulphide bridge in the absence of any other associated domains. The unbonded cysteines could also serve as redox sensors that regulate the binding of ligands by this domain.

The first structure of the YARHG domain gives us the opportunity to look at the reason that the YARHG motif is conserved among members of the family. Detailed examination of the structure shows that the most conserved sequence-region corresponds to the structural region that contains a rather unusual feature, *i.e.* that of crossing loops. The underlying loop connects helices H8 and H9 (Figure 1), whereas the next loop, between H9 and H10, crosses over the underlying loop. The YARHG sequence motif (the actual sequence in lpg2210 is YAQYG) maps onto the C-terminal part of H8 with the conserved Gly residue forming its C-terminal cap. The other conserved residues probably contribute to the stabilization of this structural feature. In particular, the small residue (Ala) is packed against several conserved aromatic residues ‘upstream’ of the YARHG motif (F308, F317, W322 and Y323). The sequence and structural conservation of this region suggest again that it might contribute to the binding of a yet unknown specific ligand of this family.

**Figure 4:**
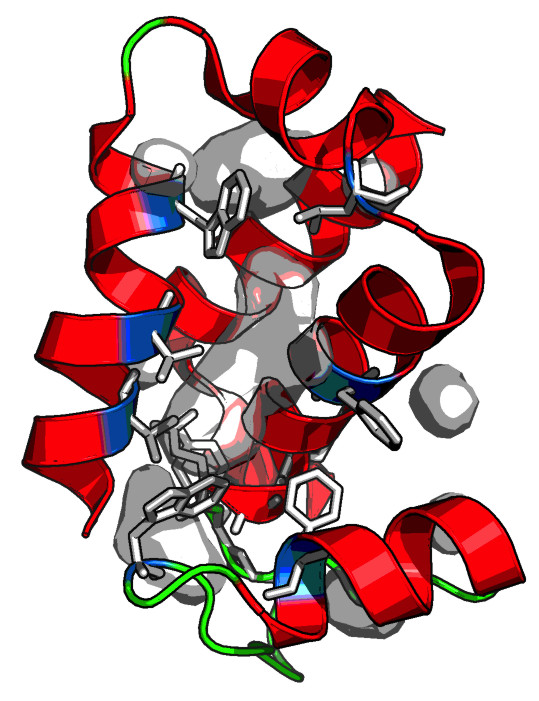
**Ribbon representation of the proposed ligand-binding pocket of the YARHG domain.** The structure [PDB:4g2a] was analyzed in PyMOL for potential ligand binding pockets by using a 2x solvent radii probe (2.8 Å).

Three lines of evidence support the role of the YARHG as a ligand-binding domain that specializes in sensing extracellular ligands: 1) The YARHG domain is located in a predicted extracellular position with intracellular signaling domains such as a S/T protein kinase domain and three distinct kinds of intracellular Zn-ribbon domains. This architectural theme has been previously observed in several sensory proteins [8] and by analogy suggests that the binding of the ligand is communicated to intracellular domains; 2) The current structure reveals that the alpha-helical bundle adopted by the YARHG domain (Figure 4) is rather open in its configuration. This suggests that it would potentially facilitate interactions with a small molecule via this open pocket. Probing the pocket using 2 solvent radii binding helps better define this potential ligand-binding site; 3) Examination of the residues lining this pocket shows the presence of an unusual overrepresentation of exposed hydrophobic residues that are not involved in stabilizing the core via hydrophobic packing. This observation suggests they are likely to provide an interface for interacting with a hydrophobic ligand via solvent exclusion (Figure 4). In particular the association of the YARHG domain with extracellular peptidoglycan-binding domains such as the bacterial SH3 and PASTA domains is suggestive of a role for the YARGH domain in binding a moiety in proximity with peptidoglycan, such as the hydrophobic outer membrane lipid or lipopolysaccharides. In this capacity it might help anchor a variety of extracellular peptidase domains to the cell-surface.

### Sequence analysis

**Figure 5:**
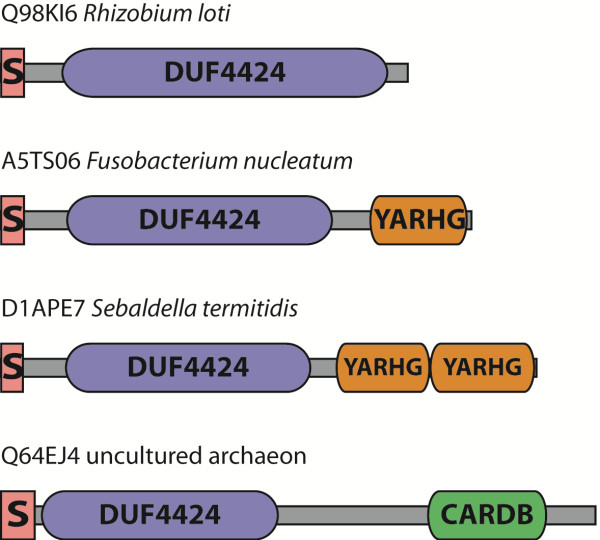
**Representative domain architectures of sequences carrying a DUF4424 domain.** Four different domain compositions or architectures are found amongst the sequences in the DUF4424 family. The signal-peptide is shown in pink, the DUF in blue and the associated, YARHG, domain in yellow, the CARDB domain in green.

To investigate further the potential functions of the DUF4424 domain we examined all the differing multiple domain architectural contexts in which the domain is found, as shown in Figure 5. The family DUF4424 in the current release of Pfam (27.0) consists of 165 sequences from UniProtKB [9]. Almost all proteins in the DUF4424 family carry a predicted signal peptide at the N-terminus indicating that they are secreted proteins. Some 70% carry only the DUF4424 domain, with no other associated domains. The remaining 30% have an associated YARHG domain at their C-terminal end. The proteins range in length between approximately 300 and 360 amino acids. There is one sequence, [UniProtKB:Q64EJ4], that is longer than the average, and, although not carrying a YARHG domain, has a CARDB domain at the C-terminus. The CARDB domain is related to bacterial cell-adhesion.

The sequence alignment of the DUF4424 domain does not suggest any strongly conserved short motifs suggestive of a particular function, and is shown for a representative set in Figure 3A.

### Species distribution

Pfam family DUF4424 is found predominantly in Gram-negative bacteria, in particular in Fusobacteria and Proteobacteria species, where it is found in Alpha-, Beta-, Gamma- and Delta-proteobacteria. Many of these species make up part of the natural gut and oral flora of a human, but can also be involved in human disease. For example, *Fusobacterium nucleatum* is an oral bacterium, indigenous to the human oral cavity, that plays a role in periodontal disease. The organism is commonly recovered from different monomicrobial and mixed infections in humans and animals. It is a key component of periodontal plaque due to its abundance and its ability to co-aggregate with other species in the oral cavity [10]. The sequence containing the CARDB domain came from a genomic analysis of deep-sea sediments [11] and is annotated as being of archaeal origin.

### Genomic context

In bacterial families it has been shown that analysis of the gene-neighborhood can give hints about the function of a protein family [12]. Our analysis of genomic contexts using MicrobesOnline [13] and STRING [14] did not show any clearly recurrent associations with other genes that might give a hint towards function.

## Conclusions

In this work we present the novel structure of the lpg2210 protein from the bacterium *Legionella pneumophila.* The structure confirms the domain organization that was inferred through careful sequence analysis in the Pfam database. The N-terminal domain was found to share structural similarity to a variety of peptidase-associated domains. Based on these structural similarities we suggest that the lpg2210 protein is a part of a multiple-component enzyme, possibly pairing with a catalytic partner. Our analysis is also suggestive of a role for the YARGH domain in binding a moiety in proximity with peptidoglycan. This could be a hydrophobic outer membrane lipid or lipopolysaccharide. In this capacity it might help anchor a variety of extracellular peptidase domains to the cell-surface.

## Competing interests

The authors declare that they have no competing interests.

## Authors’ contributions

PC and AGB wrote the manuscript; PC, RYE, AGB, RDF and YC carried out the sequence-analysis; LJ, AG, DD, QP, HLA carried out strucuture-determination; LA and AGM performed sequence- and structural-analyses. All authors read and approved the final manuscript.

## Supplementary Material

Additional file 1 **Experimental Details [PDB:4g2a] [UniProtKB:Q5ZTF2].** This section contains the detailed Materials and Methods with an appropriate Table of results for the determination of the structure under investigation. Click here for file
