# Supplementary material for: Two Pfam protein families characterized by a crystal structure of protein lpg2210 from Legionella pneumophila
Source: BMC Bioinformatics. 2013 Sep 3;14:265. doi: 10.1186/1471-2105-14-265 (PMC3848476; doi:10.1186/1471-2105-14-265)
Supplement: Additional file 1 — Experimental Details [PDB:4g2a] [UniProtKB:Q5ZTF2]. This section contains the detailed Materials and Methods with an appropriate Table of results for the determination of the structure under investigation. [file 1471-2105-14-265-S1.docx]

**EXPERIMENTAL DETAILS [PDB:4g2a] [UniProtKB:Q5ZTF2]**

**Materials and Methods**

**Data collection, structure solution, and refinement**

All X-ray diffraction data were collected at the Stanford Synchrotron Radiation Lightsource (SSRL) on beamline 13-2 Data sets were collected at 100 K using a Dectris Pilatus 6M Pixel Array Detector. X-ray diffraction data were collected from a single crystal at wavelengths corresponding to the high energy remote (λ_1_) and inflection (λ_2_), and peak (λ_3_) of a three-wavelength selenium multi-wavelength anomalous diffraction (MAD). The data were integrated and scaled using the XDS and XSCALE programs respectively (Kabsch, 1993, 2010). Data statistics are summarized in Table 1. The selenium substructures for the three proteins were solved with SHELXD (Schneider and Sheldrick, 2002) and the MAD phases were refined with *autoSHARP* (Vonrhein *et al.,* 2007) with a mean figure of merit of 0.31. Iterative automated model building was performed with at an Arp/Warp (Langer *et al.*, 2008) at a resolution of 2.33 Å from density-modified electron density. Model completion was performed using the interactive computer-graphics program COOT (Emsley and Cowtan, 2004) and MAD-phase-restrained refinement was accomplished using the program BUSTER version 2.10.0 (Bricogne *et al.,* 2011, Smart *et al*., 2012) at a resolution of 2.33 Å.

**Validation and deposition**

The quality of the crystal structure was analyzed using the JCSG Quality Control server (<http://smb.slac.stanford.edu/jcsg/QC/>). This server verifies: the stereochemical quality of the model using AutoDepInputTool,(Yang *et al*., 2004), MolProbity, (Chen *et al.*, 2010), and WHATIF 5.0 (Vriend, 1990); agreement between the atomic model and the data using SFcheck 4.0, (Vaguine, et al., 1999), and RESOLVE (Terwilliger, 2004); the protein sequence using CLUSTALW (Chenna *et al*., 2003) ; atom occupancies using MOLEMAN2.0 (Kleywegt, 1997); and consistency of NCS pairs. It also evaluates differences in Rcryst/Rfree, expected Rfree/Rcryst, and maximum/minimum B-values by parsing the refinement log-file and PDB header. Protein quaternary structure analysis used the EBI PISA server (Krissinel and Henrick, 2007). Figures 1, 2 and 3 were prepared with PyMOL (Schrödinger LLC). Atomic coordinates and experimental structure factors at have been deposited in the PDB and are accessible under the code 4G2A.

**References**

Bricogne G., Blanc E., Brandl M., Flensburg C., Keller P., Paciorek W., Roversi P, Sharff A., Smart O.S., Vonrhein C., Womack T.O. (2011). BUSTER version 2.1.0 Cambridge, United Kingdom: Global Phasing Ltd.

Chen VB, Arendall WB, 3rd, Headd JJ, Keedy DA, Immormino RM, Kapral GJ, Murray LW, Richardson JS, Richardson DC. MolProbity: all-atom structure validation for macromolecular crystallography. Acta crystallographica Section D, Biological crystallography 2010;66(Pt 1):12-21.

Chenna R, Sugawara H, Koike T, Lopez R, Gibson TJ, Higgins DG, Thompson JD. Multiple sequence alignment with the Clustal series of programs. Nucleic acids research 2003;31(13):3497-3500.

Cruickshank DW. Remarks about protein structure precision. Acta crystallographica Section D, Biological crystallography 1999;55(Pt 3):583-601.

Diederichs K, Karplus PA. Improved R-factors for diffraction data analysis in macromolecular crystallography. Nature structural biology 1997;4(4):269-275.

Emsley P, Cowtan K. Coot: model-building tools for molecular graphics. Acta Cryst D 2004;60(Pt 12 Pt 1):2126-2132.

Kabsch W. Automatic Processing of Rotation Diffraction Data from Crystals of Initially Unknown Symmetry and Cell Constants. J Appl Cryst 1993;26:795-800.

Kabsch W. XDS. Acta Crystallogr D Biol Crystallogr. 2010 Feb;66(Pt 2):125-32. doi: 10.1107/S0907444909047337. Epub 2010 Jan 22. PubMed PMID: 20124692; PubMed Central PMCID: PMC2815665.

Kleywegt GJ. Validation of protein models from Calpha coordinates alone. J Mol Biol 1997;273(2):371-376.

Krissinel E, Henrick K. Inference of macromolecular assemblies from crystalline state. J Mol Biol 2007;372(3):774-797.

Langer G, Cohen SX, Lamzin VS, Perrakis A. Automated macromolecular model building for X-ray crystallography using ARP/wARP version 7. Nat Protoc.2008;3(7):1171-9. doi: 10.1038/nprot.2008.91. PubMed PMID: 18600222;PubMedCentral PMCID: PMC2582149.

Smart, O. S., Womack, T. O., Flensburg, C., Keller, P., Paciorek, W., Sharff, A., Vonrhein, C. & Bricogne, G. (2012). Exploitingstructure similarity in refinement: automated NCS and target-structure restraints in BUSTER. Acta Cryst. D68, 368-380

Terwilliger, T.C. and Berendzen, J. (1999) “Automated MAD and MIR structure solution” Acta Cryst D55, 849-861.

Vaguine AA, Richelle J, Wodak SJ. SFCHECK: a unified set of procedures for evaluating the quality of macromolecular structure-factor data and their agreement with the atomic model. Acta crystallographica Section D, Biological crystallography 1999;55(Pt 1):191-205.

Vriend G. WHAT IF: a molecular modeling and drug design program. Journal of molecular graphics 1990;8(1):52-56, 29.

Weiss MS. Global indicators of x-ray data quality. Journal of Applied Crystallography 2001;34:130-135.

Yang H, Guranovic V, Dutta S, Feng Z, Berman HM, Westbrook JD. Automated and accurate deposition of structures solved by X-ray diffraction to the Protein Data Bank. Acta crystallographica Section D, Biological crystallography 2004;60(Pt 10):1833-1839.

**Table 1** Summary of crystal parameters, data collection, and refinement statistics for [UniProtKB:Q5ZTF2] from *Legionella pneumophila* subsp. pneumophila str. philadelphia 1 [PDB:4g2a]

| Space group | | P2_1_2_1_2_1_ | | | | |
| --- | --- | --- | --- | --- | --- | --- |
| Unit cell parameters | | a=37.95 Å b=87.72 Å c=99.77 α=β= γ=90º | | | | |
| **Data collection** | | λ_1_ MADSe | | | λ_2_ MADSe | λ_3_ MADSe |
| Wavelength (Å) | | 0.9184 | | | 0.9795 | 0.9794 |
| Resolution range (Å) | | 43.36-2.77 | | | 43.36-2.68 | 43.36-2.33 |
| No. of observations | | 30,268 | | | 32,945 | 50,142 |
| No. of unique reflections | | 8743 | | | 9590 | 14,552 |
| Completeness (%) | | 98.1 (99.2)^a^ | | | 97.5 (87.2)^a^ | 98.3 (98.5)^a^ |
| Mean I/σ(I) | | 11.9 (2.0)^a^ | | | 12.2 (1.7)^a^ | 13.0 (1.9)^a^ |
| R_sym_ on I (%) † | | 11.9(68.2)^a^ | | | 10.8 (70.2)^a^ | 9.0(68.5)^a^ |
| R_meas_ on I (%)‡ | | 14.0 (79.9)^a^ | | | 12.7 (84.4)^a^ | 10.6 (80.5)^a^ |
| Highest resolution shell (Å) | | 2.84-2.77 | | | 2.75-2.68 | 2.39-2.33 |
| **Model and refinement statistics** | | | | | | |
| Resolution range (Å) | 43.36-2.33 | | | Data set used in refinement | | λ_3_ MADSe |
| No. of reflections (total) | 14,523 | | | Cutoff criteria | | \|F\|>0 |
| No. of reflections (test) | 753 | | | R_cryst_ ¶ | | 0.168 |
| Completeness (%total) | 97.8 | | | R_free_ § | | 0.222 |
| **Stereochemical parameters** | | | | | | |
| Restraints (RMSD observed) | | | | | | |
| Bond angle (°) | | | 1.07 | | | |
| Bond length (Å) | | | 0.009 | | | |
| Average isotropic B-value (Å^2^) | | | 42.49 | | | |
| ESU †† based on R_free_ (Å) | | | 0.230 | | | |
| Protein residues/atoms | | | 321/2596 | | | |
| Water /Ions | | | 160/14 | | | |

^a^highest resolution shell

†R_sym_ = Σ|I_i_-<I_i_>| / Σ|I_i_| where I_i_ is the scaled intensity of the i^th^ measurement and <I_i_> is the mean intensity for that reflection.

‡R_meas_ is the redundancy-independent Rsym. ([Diederichs & Karplus, 1997](#_ENREF_16); [Weiss, 2001](#_ENREF_56)).

¶R_cryst_ = Σ| |F_obs_|-|F_calc_| | / Σ|F_obs_| where F_calc_ and F_obs_ are the calculated and observed structure factor amplitudes, respectively.

§R_free_ = as for R_cryst_, but for 5.1 % of total reflections chosen at random and omitted from refinement.

†† ESU = Estimated overall coordinate error ([Cruickshank, 1999](#_ENREF_14))
